# A clinical study of peripherally inserted central catheter-related venous thromboembolism in patients with hematological malignancies

**DOI:** 10.1038/s41598-022-13916-5

**Published:** 2022-06-14

**Authors:** Jing Yue, Ya Zhang, Fang Xu, Ai Mi, Qiaolin Zhou, Bin Chen, Lin Shi

**Affiliations:** Department of Hematology, Mianyang Central Hospital, School of Medicine, University of Electronic Science and Technology of China, Mianyang, 621000 China

**Keywords:** Cancer, Diseases, Health care, Medical research, Oncology, Risk factors

## Abstract

This study aimed to explore the risk factors of peripherally inserted central catheter (PICC)-related venous thromboembolism (CRT) in patients with hematological malignancies and the predictive ability of the thrombotic risk assessment models (RAMs). The clinical data of the 117 eligible patients with hematological neoplasms at Mianyang Central Hospital with PICC from May 2018 to May 2020 were analyzed in this retrospective study. Thrombosis risk scores were calculated in patients with image-confirmed PICC-related thromboembolism. CRT occurred in 19 cases. Compared to the CRT-free group, the CRT group was older and showed higher body mass index (BMI), leukocyte count level, and the prevalence of diabetes mellitus. Multivariable logistic regression analysis showed that BMI (*P* = 0.03) was a significant risk factor for CRT. The area under the receiver operating characteristic curve for the Caprini scale (*P* = 0.01) was higher than that of the modified Wells scale (*P* = 0.94), the revised Geneva scale (*P* = 0.83), Padua scale (*P* = 0.59), and Michigan scale (*P* = 0.80). The sensitivity and specificity for the Caprini scale, Padua scale, modified Wells scale, the revised Geneva scale, and Michigan risk score were 63.3%/73.7%, 100%/0.00%, 95.9%/5.3%, 31.6%/73.7%, and 1.0%/99.0%, respectively. Caprini RAM had a better predictive ability for CRT in patients with hematological malignancies. Michigan risk score may not be better than Caprini RAM in this population.

## Introduction

A peripherally inserted central catheter is widely used for patients with hematologic malignancies due to simple insertion procedures, long dwell time, and easy maintenance protocol. It is a safe, convenient, economical, and less painful infusion method. A PICC is suitable for infusion of chemotherapeutic agents, nutrition, blood products, high concentration medicine, and other cytotoxic agents to avoid peripheral blood vessel impairment, tissue necrosis, or local phlebitis caused by drug extravasation^1–3^. One of the foremost complications related to PICC is catheter-related vein thrombosis (CRT)^4^, with an incidence of 0%–71.9%^3,5,6^. In addition to pain and discomfort related to the catheter^7^, CRT may result in extra financial burden, treatment delay or interruption, and fatal consequences^8^. For now, however, there is no generally accepted risk prediction model for CRT. For patients with hematological malignancies, biological characteristics of tumors are baseline factors leading to CRT. Compared to solid tumors, concomitant severe thrombocytopenia, coagulopathy, and bone marrow suppression make the application of prophylactic measures challenging for CRT. Therefore, the risk prediction of CRT in hematologic malignancy patients is rather complicated. Thus, it is necessary to investigate the CRT-related risk factors and prediction models for hematology patients.

This retrospective study reviewed hemato-oncology patients with PICC insertion and explored the risk factors of CRT in hematological cancer patients and examined the predictive value of the traditional thrombotic risk assessment models for CRT.

## Methods

### Study population

This retrospective study of 117 patients with hematological malignancies having PICC insertion at Mianyang Central Hospital (Mianyang, China) from May 2018 to May 2020. The malignancies, including leukemia, malignant lymphoma, myelodysplastic syndrome, and multiple myeloma, were confirmed through auxiliary examinations, such as bone marrow morphology, bone marrow flow cytometry, and lymph node biopsy. The inclusion criteria were as follows: diagnosed with a hematological tumor and needed PICC catheterization. The exclusion criteria were as follows: patients aged < 14-years-old and lost to during the follow-up. The study was approved by the Ethics Committee of Mianyang Central Hospital (approval number: S2021077). Written informed consent was obtained from parents or guardians of the participants who are less than 16 years old involved in the study.

### Data collection

Patient-related characteristics at baseline and several potential risk factors of CRT reported in the literature were collected: general conditions (gender, age, and body mass index (BMI)), disease states and comorbidities (hematological tumor type, smoking, drinking, diabetes, hyperlipidemia, infection status, and history of thrombosis), biochemical indexes (platelets, fibrinogen concentration, prothrombin time, activated partial thromboplastin time, and kidney function), catheter-related data (vein entry and laterality), exposure to blood products and special medications (blood component, human serum albumin, plasma fibrinogen, erythropoietin, and parenteral nutrition). These were classified as CRT groups and no CRT groups based on whether or not PICC-related venous thromboembolism was formed.

### Thrombosis risk assessment scales for reference

Several risk scoring tools^9–13^ were applied to assess the thrombosis risk before PICC catheterization in all participants, and the cumulative score for each patient was then calculated.

### Detecting venous thrombosis

This research defined CRT as thrombosis involving the peripherally inserted central catheter-associated upper extremity deep vein thrombosis and mural thrombosis. Symptoms include redness, swelling, heat, and pain at the catheter arm, intravenous transfusion obstacle, or the catheter indwelling time for over a year. CRT was confirmed by vascular ultrasound.

### PICC insertion and maintenance

All PICC catheterizations had been operated on under ultrasound guidance by an experienced and qualified professional nurse. All PICCs used in this study were single-lumen catheters (PowerPICC, Bard Access Systems, Inc., Salt Lake City, Utah, USA) with a model of 4-French and were routinely maintained by PICC specialist nurses using sterile technique weekly. A 45% catheter-to-vein ratio limit was used when inserting PICC devices. All the procedures were performed in accordance with the PICC specifications. PICC tip position at the cavoatrial junction is all confirmed via X-ray.

### Statistical analysis

Categorical variables were expressed as frequencies and percentages and compared between the groups using the chi-square test. Continuous variables were presented as mean ± standard deviation. An independent-sample t-test or Mann–Whitney U test was used to compare the difference between the groups. A binary logistic regression model was utilized to examine the association between risk factors and CRT . Receiver operating characteristic (ROC) curves were plotted according to the sensitivity and specificity of RAMs, and the area under the curve (AUC) and 95% confidence interval (CI) were also calculated. *P* < 0.05 was considered statistically significant. Statistical analysis was done with SPSS software (IBM Corp, Released 2016, IBM SPSS Statistics for Windows, version 24.0, Armonk, NY, USA).

### Ethical approval and consent to participate

Written informed consent prior to and regarding the treatment protocol was obtained from all patients analyzed in the present study. The procedures used in this study adhere to the tenets of the Declaration of Helsinki.

### Consent for Publication

Informed consent was obtained from all individual participants included in the study.

## Results

### General characteristics of CRT and no CRT cases

A total of 117 participants, including 63 men (53.4%) and 54 women (45.8%), with a median age of 54 (range: 15–90 years), were included in this study. 19/117 (16.2%) patients were verified as developing PICC-related venous thromboembolism. Of these cases, 14 were mural thrombosis and 5 were deep venous thrombosis. Compared to no CRT patients, CRT patients presented significant differences in median age(*P* = 0.04), the prevalence of diabetes mellitus(*P* = 0.04), BMI(*P* = 0.007), white blood cell count(*P* = 0.04), and prothrombin time(*P* = 0.03). On the other hand, no differences were detected in gender, disease states, comorbidities, biochemical indexes, catheter-related data, exposure to blood products, and special medications (Table 1).

**Table 1:** Clinical features of hematological malignancy patients with and without CRT.

Variables	Patients without CRT (n = 98)	Patients with CRT (n = 19)	*P-*value
Age, years	51.2 ± 17.6	60.2 ± 15.0	0.039*
Gender (Male/Female), n	54/44	9/10	0.536
BMI, kg/m^2^	22.6 ± 4.2	25.68 ± 4.1	0.007*
Cancer			
Acute leukemia, n (%)	50 (51.00)	8 (42.10)	N/A
Lymphoma, n (%)	37 (37.80)	9 (47.40)	
Plasma cell disease, n (%)	4 (4.10)	2 (10.50)	
Other hematological malignancies, n (%)	7 (7.10)	0 (0.00)	
**Cell origin**			
Myeloid, n (%)	42 (42.9)	5 (26.3)	0.335
B cell, n (%)	49 (50.0)	13 (68.4)	
T/NK cell, n (%)	7 (7.1)	1 (5.3)	
Smoking, n (%)	20 (20.4)	3 (15.8)	0.762
Drinking, n (%)	12 (12.2)	2 (10.5)	1.000
Hypertension, n (%)	6 (6.1)	4 (21.1)	0.056
Diabetes, n (%)	8 (8.2)	5 (26.3)	0.037*
Hyperlipidemia, n (%)	4 (4.1)	1 (5.3)	1.000
infection(grade ≥ 3) , n (%)	55 (56.1)	6 (31.6)	0.050
Blood products transfusion, n (%)	83 (84.7)	14 (73.7)	0.315
Erythropoietin use, n (%)	4 (4.1)	0 (0.0)	1.000
Parenteral nutrition support, n (%)	49 (50.0)	7 (36.8)	0.293
Catheter placement(left/right), n	45/53	9/10	1.000
**Vessel puncture**			
Basilic vein, n (%)	81 (82.7)	14 (73.7)	N/A
Median cubital vein, n (%)	13 (13.3)	4 (21.1)	
Saphenous vein, n (%)	4 (4.1)	1 (5.3)	
**Biochemical indexes of PICC catheterization**			
WBC count, × 10^9^/L	16.58 ± 44.49	24.20 ± 33.55	0.042*
Platelet, × 10^9^/L	118.5 ± 105.5	133.5 ± 69.9	0.566
Fibrinogen, g/L	3.3 ± 1.4	3.3 ± 1.29	0.950
PT, s	12.6 ± 1.2	12.0 ± 1.5	0.025*
APTT, s	29.7 ± 6.5	27.2 ± 3.9	0.141
Creatinine, mmol/L	68.7 ± 81.7	75.3 ± 52.3	0.744
Glomerular filtration rate, mL/min/1.7	74.1 ± 22.5	74.1 ± 27.2	0.994
Creatinine clearance, mL/min	106.4 ± 41.3	96.3 ± 30.6	0.356

*BMI* Body mass index, *PICC* Peripherally inserted central catheter, *CRT* Peripherally inserted central catheter -related venous thromboembolism, *WBC* White blood cell, *PT* Prothrombin time, *APPT* Activated partial thromboplastin time.

*Statistically significant.

### Multivariate correlations between risk factors and CRT

Univariate analysis showed that age, BMI, white blood cell count, prothrombin time, the prevalence of diabetes mellitus, the incidence of hypertension, and the rates of grade 3 or 4 infections were potential predictive risk factors. These factors were entered in a binary logistic regression analysis. Results showed that BMI (OR = 1.315, 95% CI: 1.033–1.674, *P* = 0.03) and prothrombin time (OR = 0.279, 95% CI: 0.097–0.802, *P* = 0.02) were the independent risk factors for CRT (Table 2).

**Table 2:** Risk Factors associated with PICC-associated venous thromboembolism (multivariate logistic regression analysis).

	OR	95% CI	*P*-value
Age	1.032	0.964–1.105	0.361
BMI	1.315	1.033–1.674	0.026
Diabetes	0.306	0.039–2.397	0.260
Hypertension	0.188	0.012–2.858	0.229
Infection (grade ≥ 3)	1.538	0.325–7.284	0.588
WBC count	0.998	0.985–1.010	0.707
Prothrombin time	0.279	0.097–0.802	0.018

*BMI* Body mass index, *PICC* Peripherally inserted central catheter, *WBC* White blood cell.

Variables that had a *P*-value < 0.10 (age, BMI, diabetes, hypertension, PICC-related infections, WBC) in univariable analysis were selected for inclusion in the multivariable model.

### Predictive capability of different risk assessment scales for CRT

**Figure 1:**
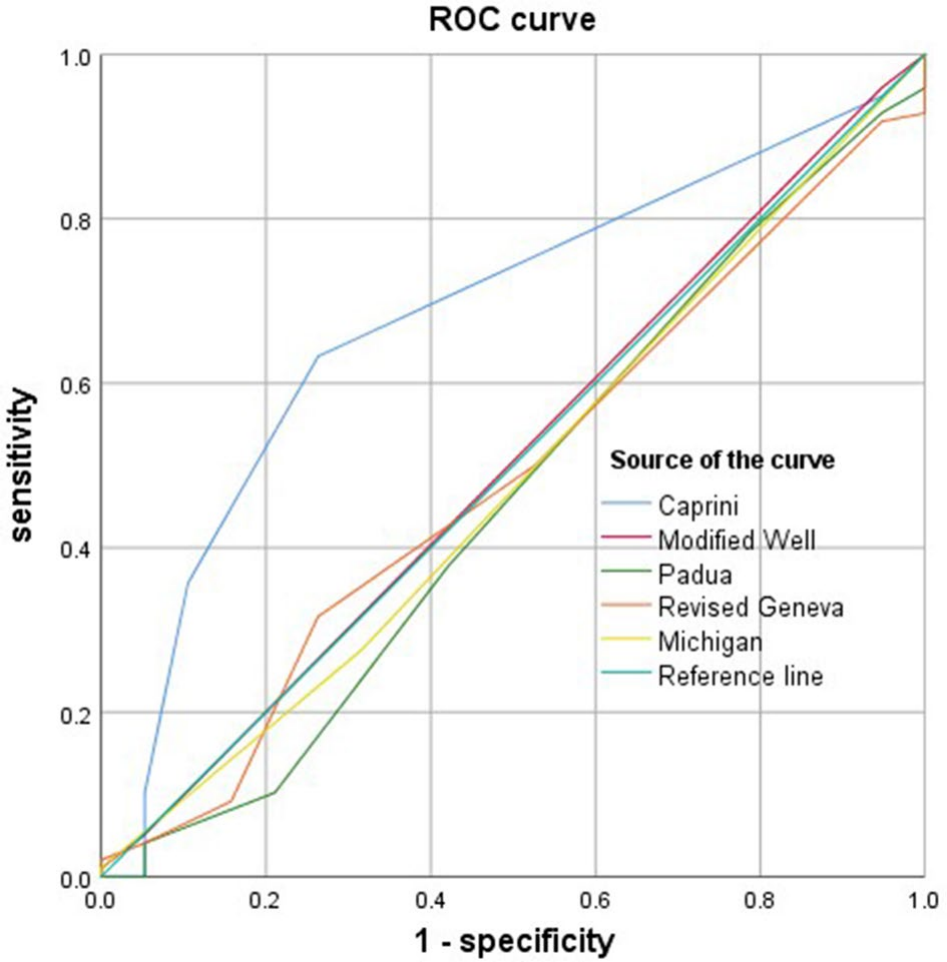
ROC curves of the five different thrombosis risk assessment scales.

Patients’ risk scores were calculated using the Caprini scale, Padua scale, modified Wells scale, revised Geneva scale, and Michigan risk score. Subsequently, the ROC curves were plotted and used to assess the validity of different thrombosis risk assessment scales of CRT. The AUC value for Caprini scale (0.683, 95% CI: 0.557–0.808, *P* = 0.01) was higher than that for modified Wells scale (0.505, 95% CI: 0.362–0.649, *P* = 0.94), revised Geneva scale (0.485, 95% CI: 0.345–0.624, *P* = 0.83), Padua scale (0.461, 95% CI: 0.312–0.610, *P* = 0.59), and Michigan scale (0.481, 95% CI: 0.338–0.625, *P* = 0.80) (Fig. 1). The sensitivity and specificity for Caprini scale, Padua scale, modified Wells scale, the revised Geneva scale, Michigan risk score were 63.3%/73.7%, 100%/0.00%, 95.9%/5.3%, 31.6%/73.7%, and 1.0%/99.9%, respectively. Interestingly, the Caprini assessment scale was more accurate than the Padua scale, modified Wells scale, the revised Geneva scale, and Michigan risk score for predicting CRT; 5.5 was the best cutoff point for Caprini, with a sensitivity of 0.633 and a specificity of 0.737 (Table 3).

**Table 3:** Comparison of the five different thrombosis risk assessment scales in predicting the occurrence risk of CRT in patients with hematological cancers.

Scales	AUC	Sensitivity (%)	Specificity (%)	Youden index (%)
Caprini	0.557–0.808	63.3	73.7	37.0
Padua	0.312–0.610	100.0	0.0	0.0
Modified Wells	0.362–0.649	95.9	5.3	1.2
Revised Geneva	0.345–0.624	31.6	73.7	5.3
Michigan	0.338–0.625	1.0	99.0	1.0

## Discussion

CRT is defined as a blood clot formed around the external portion of the catheter or internal portion of the vascular walls. Consequently, the risk of VTE may be higher for hematological malignancy patients than for those with solid tumors; this phenomenon could be attributed to multiple factors, such as the nature of neoplasm, aging population, and co-morbidities. Hematologic tumor patients are more likely to have CRT than solid tumors, with a rate of 1.9–18.7%^1,14,15^. In this research, 12.0% of patients were the peripherally inserted central catheter-associated mural thrombosis and 4.2% were deep venous thrombosis, which is consistent with the findings from previous studies. CRT could lead to phlebitis, vascular stenosis, pulmonary embolism, and could even be life-threatening^16^. On the other hand, CRT in hemato-oncology patients has specific characteristics. The practical implementation of the thromboprophylaxis measures for CRT is complicated in hematological cancer patients, who have a high risk of hemorrhage resulting from thrombocytopenia, coagulopathy, or bone marrow suppression.

The present study explored the CRT-related clinical risk factors. Advanced age, BMI, history of diabetes mellitus, hypertension history, leukocyte count, prothrombin time, and grade 3 or 4 infections were identified as putative risk factors for patients with hematologic tumors. Various studies supported different clinical features to be CRT related risk factors. Some studies reported that high BMI, high PLT count, non-O blood group, high level of triglycerides, catheter to vein ratio > 0.45, more than one attempt for PICC insertion, and fluoropyrimidine containing chemotherapy are potential risk factors for CRT events^17–21^. However, all of the above studies mainly focused on the solid tumor population^22^. Also, there was a lack of reliable evidence from hemato-oncology patients. Scrivens et al. demonstrated that PICC type was associated with the incidence and rate of PICC-associated venous thromboembolism in hematologic malignancies^22^. Another study reported that no feature was predictable for a high risk of catheter-related thrombotic complications in hematology patients^1^. Univariate analysis showed that age, BMI, the prevalence of diabetes mellitus and hypertension, prothrombin time, leukocyte count, and infections > grade 3 or 4 severity were potential predictive risk factors in hematological cancer patients in this study. However, multivariate logistic regression analysis showed that BMI and prothrombin time were the independent risk factors for CRT. Notably, the prothrombin time was within the normal range in both groups. The clinical significance for CRT in patients with hematological malignancies was limited. This finding is consistent with the previously reported clinical investigations that BMI^19,23^ may be a crucial risk predictor for CRT in patients with hematological cancer. Considering blood hypercoagulation and slow blood flow, cardiovascular diseases may occur in obese patients. Thus, adopting close vascular monitoring, positive exercises, diet intervention, and antithrombotic prophylaxis is necessary for obese patients to reduce the risk of CRT.

Herein, no specific thrombotic risk assessment model was applied to stratify the risk of CRT in patients with hematological malignancy. Classic VTE assessment scales include Caprini, Padua, modified Wells, and the revised Geneva score. The conclusive scores were obtained from patients from General Surgery, Internal Medicine, outpatients, or intensive care unit (ICU), respectively^9,11,12,24^. Few studies have attempted to apply the classic VTE risk models to assess the thrombosis risk of PICC^25,26^. The Michigan risk score is the first risk model for CRT prediction in recent years. Nonetheless, the model also encompassed patients admitted to the General Medicine ward or ICU as the main research subjects. This study compared the potential predictive power of CRT risk in hemato-oncology patients, using Caprini, Padua, modified Wells, the revised Geneva and Michigan risk score. Firstly, in this study, the Michigan model was not superior to the other four classic models in this study. The predictor variables in the Michigan risk score include the presence of another CVC when index PICC is placed, WBC count at the time of PICC insertion, active cancer, number of PICC lumens, and history of venous thromboembolism^13^. Herein, only a single-lumen catheter was used. Supposedly, in hematologic malignancy patients, WBC count may be affected by many factors. Hence, WBC count may not be a predictive variable sensitive in the hematology patient as in the General Medicine ward or the ICU patients. Also, the Michigan risk score was obtained from the Western populations. This might indicate that the characteristics and risk model of CRT may be different based on the underlying diseases. Secondly, the current study identified that the Caprini assessment scale had a greater predictive value in the assessment of CRT than other models. This finding was consistent with that of Feng et al., wherein the Caprini scale was used for high-risk screening of the CRT in cancer patients, and the AUC value was 0.636 (95% CI: 0.590–0.680; *P* < 0.001)^25^. Unlike Feng et al., this study compared the predictive value of five different risk assessment scales simultaneously. Compared to the Padua scale, the modified Wells scale, the revised Geneva, and Michigan risk scale, the Caprini scale has better sensitivity and specificity in the risk prediction of CRT in patients with hematology tumors.

Nevertheless, the present study has several limitations. First, it was a retrospective study. Second, the study was conducted at a single research institution, and the population size was small. All PICCs used in this study were single-lumen catheters. Due to the small number of patients, the authors were unable to evaluate the putatively related risk factors reported in the literature, such as PICC brand, the type of PICC, and chemotherapy regimen. Thus, large, multicenter, prospective studies are necessary to substantiate these findings in the future. Finally, some patients with PICC-related asymptomatic venous thromboembolism during the study period were not included. A prospective study assessing the asymptomatic CRT samples by a regular vascular examination is essential.

## Conclusion

BMI is a significant risk factor for CRT in patients with hematological cancer. It is necessary to strengthen monitoring and intervention to reduce the risk of CRT for patients with high BMI. Caprini thrombosis risk assessment model has a better predictive value for CRT than other tools and could be used as an effective risk prediction tool in patients with hematologic malignancies.

## Data Availability

The datasets used and analyzed during the current study are available from the corresponding author on reasonable request.
